# Supplementary figures and images for: Wave generator as an alternative for classic and innovative wave transmission path vibration mitigation techniques
Source: PLoS One. 2021 Jun 2;16(6):e0252088. doi: 10.1371/journal.pone.0252088 (PMC8171992; doi:10.1371/journal.pone.0252088)

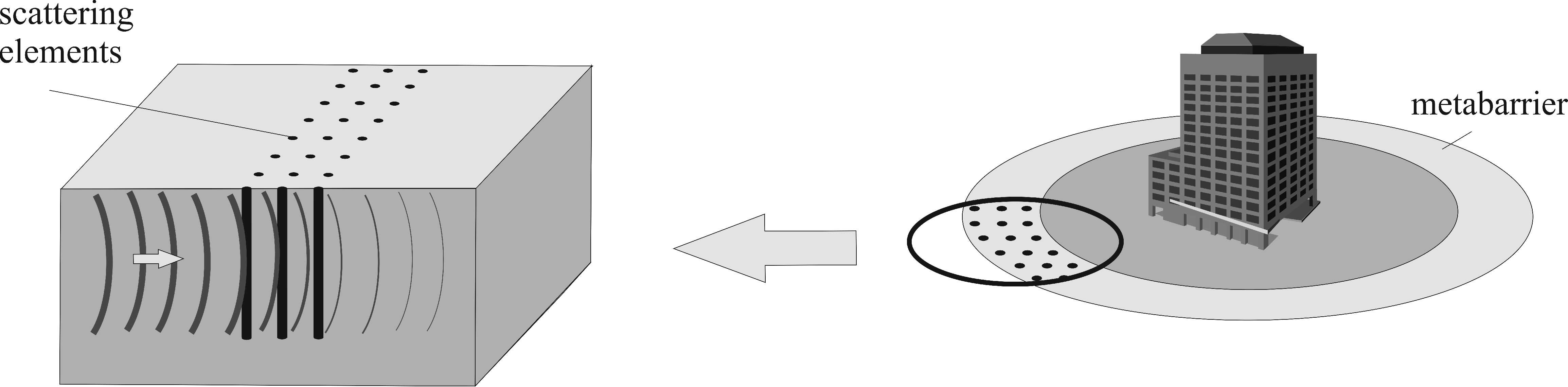

Supplement: S2 Fig — (TIF) [file pone.0252088.s002.tif]
