# Supplementary material for: Wave generator as an alternative for classic and innovative wave transmission path vibration mitigation techniques
Source: PLoS One. 2021 Jun 2;16(6):e0252088. doi: 10.1371/journal.pone.0252088 (PMC8171992; doi:10.1371/journal.pone.0252088)
Supplement: Attachment — Submitted filename: reviewers response.docx [file pone.0252088.s005.docx]

**Thank you for your comments, that turned out to be very helpful in improving my work and which without doubt increased the quality of the paper. The Paper has been revised according to the Reviewer’s comments. All comments with my response are listed below.**

1.      Section 1. Introduction: the last sentence: “This issue is very important in the case of underground structures (like tunnels) or structures with underground floors.” The sentence is removed in the revised version of the paper as the case of building with underground floors was not considered in the presented paper.

2.      Expression “man-made vibrations” is replaced by "anthropogenic vibrations" in the revised version of the paper.

4.      Section 3.1 formula (1): ∑𝑃𝑥 = 0 is the equilibrium condition for the infinitesimal soil element. The external load (both forces applied to the ground surface) is defined in the presented mathematical model, as the boundary condition for the selected area located on the ground surface (8) – (11).

5.      Damping ratio ksi=1%. I agree that the assumed value is close to the smallest possible damping in soil, however (Das B, Ramana G. Principles of Soil Dynamics. Stamford: Cengage Learning; 2011) gives the values of logarithmic decrement for small strains at about 0.04-0.15 for sand taken from resonant column tests (Das B, Ramana G. Principles of Soil Dynamics. Stamford: Cengage Learning; 2011, Fig. 2.20) and that gives the damping ratio ksi (lower limit) close to 1%.  However, during numerical analyses, it can be observed that generally, geometrical damping influences the vibration amplitudes much more than material damping. This second phenomenon does not influence the solution significantly.

6.      Table 1: In the presented numerical model a transversally isotropic soil medium is assumed. It gives 5 independent elasticity constants, not 2 as are usually used for isotopic model description (“Wave Fields in Real Media Wave Propagation in Anisotropic, Anelastic ...”J. M. Carcione). That is why there is no simple relationship between E and G in each direction considered. All values of Cij constants are given in the manuscript, C55 can be obtained based on SV wave velocity measurements (SV means wave propagation in x or y direction when particle movement is in z (vertical) direction), since Vsv=sqrt(c55/rho).

 

7.      Soil elastic parameters are taken from literature: Chen X, Birk C, Song C. Numerical modeling of wave propagation in anisotropic soil using a displacement unit-impulse-response-based formulation of the scaled boundary finite element method, Soil Dyn Earthq Eng 2014;65:243–55. https://doi.org/10.1016/j.soildyn.2014.06.019; Das B, Ramana G. Principles of Soil Dynamics. Stamford: Cengage Learning; 2011; Wrana B. Soil dynamics. Computation models. Kraków: Wydawnictwo Politechniki Krakowskiej; 2016.

8.      Formulas (6) and (7): the definition of AMF and AMF_av_ has been changed according to the Reviewer’s suggestion.

9.      The Reviewer’s comment: “Just for discussion and order of length: for usual buildings, 4 to 12 mm/s could be acceptable. Indeed, for sensitive devices inside the building, lower values may be required.” I agree that from the point of view of the structure’s safety 3mm/s is the lowest PPV limit for vibration-sensitive structures and the low frequency, long-lasting excitation (DIN 4150-3, BS 5228, …). In the Figure below PPV threshold values (for vibration-sensitive structures and long-lasting excitation) are presented.

PPV [mm/s]

| f [Hz] | DIN 4150-3 | EC3 | FTA |
| --- | --- | --- | --- |
| 0 | 3 | 2 | 3 |
| 10 | 3 | 2 | 3 |
| 50 | 8 | 2 | 3 |
| 60 | 8.4 | 2 | 3 |

| f [Hz] | SN 640312 |
| --- | --- |
| 0 | 3 |
| 10 | 3 |
| 30 | 3 |
| 60 | 5 |

However, the acceptable limits can be lower taking into account human comfort, a time when the load acts (day/night) and the building purpose (hospitals, laboratory, etc …). Moreover, much smaller values are acceptable for technical equipment in the laboratory.

10.  Section 4: this part of the paper has been improved according to the Reviewer’s suggestions.

11.  Reviewer’s suggestion: “To broaden the conclusion, indicate for example that we know how to obtain information on the arrival of a real earthquake (see Mexico City: <http://www.iitk.ac.in/nicee/wcee/article/9_vol7_673.pdf>). Knowing the incoming seismic signal, could we imagine propagating a counter-signal for this case?”

The solution proposed in the paper is rather addressed to anthropogenic vibrations than to earthquakes. By geotechnical works or machine exploitation, the load is applied directly to the ground surface and the surface wave is dominant (about 67% of the total energy). In such a case it is easy to select the appropriate counter-signal applied to the ground surface that gives the high level of vibration reduction. Moreover, there is no problem with energy supply in the case of anthropogenic vibrations. Theoretically, during earthquakes after the signal detection, it is also possible to select dominant frequencies of the recorded signal and select the counter-signal. Probably, it could work in numerical simulations, however, I am not convinced it could be efficient in real case of earthquake excitation, due to the complex nature of wave propagation in soil caused by earthquake excitation (P-, SH-, SV-waves reflected and refracted from the boundary surface between soil layers). A counter-signal gives the surface wave dominance. Moreover, there are usually problems with energy supply during such an incident.

12.  Figure 1: Figure 1 has been improved according to the Reviewer’s suggestions, additional figures Fig. 2b and Fig. 2c present the load applied as the function of time.

13. Section “References” surname has been changed to “Brûlé” in the revised version of the paper.
